# Defining and distinguishing early life stress, trauma, adversity, toxic and chronic stress and allostatic load: a descriptive review

**DOI:** 10.1177/14034948241260105

**Published:** 2024-08-01

**Authors:** Maarten C.C. Remmers, Rianne P. Reijs, Christian J.P.A. Hoebe

**Affiliations:** 1 Department of Social Medicine, Care and Public Health Research Institute, Maastricht University, Maastricht, Netherlands; 2 Department of Youth Health Care, Public Health Service Limburg-North, Venlo, Netherlands; 3 Department of Youth Health Care, Living Lab Public Health Mosa, Public Health Service South Limburg, Heerlen, Netherlands; 4 Department of Sexual Health, Infectious Diseases and Environmental Health, Living Lab Public Health Mosa, Public Health Service South Limburg, Heerlen, Netherlands; 5 Department of Medical Microbiology, Infectious Diseases and Infection Prevention, Care and Public Health Research Institute, Maastricht University Medical Center+, Maastricht, Netherlands

**Keywords:** Early life stress, adverse childhood experiences, childhood adversity, toxic stress, chronic stress, allostatic load, childhood trauma, descriptive review

## Abstract

**Aims:**

Various concepts are used to study the impact of stress on childhood development. These concepts are often used inconsistently or interchangeably. Our main objectives were to determine how selected stress concepts (chronic stress, toxic stress, allostatic load, early life stress, childhood adversity, childhood trauma and adverse childhood experiences; ACEs) are defined, operationalized and described, and to provide a theoretical context to aid the choice for a preferred concept in public health research.

**Methods:**

For this descriptive review, we systematically searched for literature published before 4 August 2021, on PubMed, Embase and PsycInfo. Two independent reviewers included studies. Exclusion criteria were: no systematic review, not peer reviewed, not published in English, selected stress concepts were no predetermined variable or a substantial topic in the discussion, full text was unobtainable or study described non-human or non-childhood populations. Data extraction forms were used. Descriptives were gathered, publication fields were identified through Journal Citation Reports categories, and verbatim descriptions were ordered in text and Venn diagrams.

**Results:**

Of 264 screened studies, 124 were included. ACEs, childhood adversity and childhood trauma were used most. ACEs were the main concept used most frequently (47.6%). A total of 11 of 14 public and environmental health journals used ACEs. All concepts refer to prolonged, repeated, interpersonal stress from 0 to 18 years, that can alter physiological systems. Four concepts were stressor oriented, two concepts focused on stress response and effect and one on the state of challenged homeostasis.

**Conclusions:**

**ACEs seem most fitting for public health setting, due to their operationalizability, large set of core experiences and widespread use.**

## Background

Prolonged or intense stress, or accumulation of stressors can be harmful and can disrupt healthy childhood development. Excessive stress during childhood development has been extensively studied in human [1,2] and animal studies [3,4]. The lifelong health consequences and associated healthcare costs make excessive stress a substantial public health problem [1,5]. Various concepts are used to study the impact of stress on childhood development from conception to adulthood. Commonly used concepts are Chronic Stress (CS), Toxic Stress (TS), Allostatic Load (AL), Early Life Stress (ELS), Childhood Adversity (CA), Childhood Trauma (CT) and Adverse Childhood Experiences (ACEs).

Of these concepts, AL and ACEs might be the most distinguished. AL was proposed in 1993 by McEwen and Stellar [6] to provide a model for the biological damage to the body and influence on health resulting from physiological responses to stressors over time [7-11]. TS is a term used to describe the high-risk end of the spectrum of positive, tolerable and toxic stress. ACEs were first described in the 1998 ACE-study by Felitti et al. [12] as a combined measure of CA. Felitti et al. connected abuse and household dysfunction during childhood to leading causes of death in adulthood. ELS and CT are used to describe situations of abuse and other excessive stressors during childhood as well. These concepts generally describe situations that can cause CS, making CS during childhood a relevant concept.

The psychophysiological model of stress [13,14] can be used to identify different aspects of the stress system (i.e. stress, stressor, stress response or stress effect). As a theoretical framework, this model can help organize the multitude of stress concepts. *Stress* can be defined as a state where homeostasis is being challenged [15]. Stimuli with the ability to disrupt homeostasis (i.e. a *stressor* [15]) can be diverse and can be classified as (a) reactive versus anticipatory stressors, or (b) physical versus psychological stressors [16]. Reactive stressors trigger the stress response through sensory signals of homeostatic challenges. Anticipatory stressors act through innate or conditioned programmes, which can occur without direct homeostatic disruption [17]. Physical stressors (threaten to) cause physical injury (e.g. physically aggressive behaviour or accidents), and psychological stressors (threaten to) cause psychological injury (e.g. social evaluation or bullying) [18]. Stressors can trigger a complex network of brain regions that collectively assess potential threats [19] and can set off processes that aim to protect or restore homeostasis through, for example, the nervous and humoural systems (i.e. the *stress response*) [13-15]. The stress response affects some of the most basic bodily functions, like blood pressure, the immune response and metabolism [13,14]. Subsequently, the stress response can impact physical and mental health [1,14], which can be described as the *stress effect* [15]. Focusing on different aspects of the stress system is likely to influence the operationalization of stress-related concepts in research.

Although the aforementioned concepts overlap, using differing operationalizations inconsistently or even interchangeably complicates research and counteracts finding solutions to the public health problem they pose [20-22]. Elucidating their overlap, differences and operationalizations can substantiate the choice for a concept and can help make stress research more comparable and efficient. Our main objectives were therefore to provide a theoretical context in which these concepts are used, to determine how different stress concepts are defined, operationalized and described in childhood stress literature, and to aid the choice for the most appropriate concept in public health research.

## Methods

Our descriptive review has clear similarities with a scoping review, as our main aim was to scope a body of literature and clarify key concepts [23]. Therefore the Preferred Reporting Items for Systematic Reviews and Meta-Analyses extension for scoping reviews was followed in the conduct and reporting of this review [24].

### Data sources

PubMed, PsycInfo and Embase were searched on 4 August 2021 for the following terms in titles and abstracts: (“early life stress” OR “adverse childhood experience*” OR “early life adversity” OR “early adversity” OR “early life trauma” OR “toxic stress” OR “chronic stress” OR “allostatic load”) AND (“pediatri*” OR “child*” OR “adolescen*”)). Additionally, the PubMed search included relevant MeSH terms. The search was filtered for systematic reviews and meta-analyses to obtain a feasible number of studies. When full texts were not directly available, we attempted to obtain them through other sources. The search was unrestricted in publication date or language. Finally, references of selected studies were checked for other eligible studies.

### Study selection

Studies were reviewed by two authors (MR and RR) independently for eligibility. Titles and abstracts were screened, and if additional information was needed, full texts were assessed. Exclusion criteria were: no systematic literature search and selection of studies, not peer reviewed, stress occurred in adulthood, stress concepts (adverse childhood experience(s), childhood/early (life) stress, childhood/early (life) adversity, childhood/early (life) trauma, TS, CS, AL) were no predetermined variable or substantial part of the discussion, non-English language, non-human population, or the full text was unobtainable.

Disagreements in assessment for eligibility were resolved through discussions between both reviewers and if necessary, a third author (CH). Cohen’s *κ* was calculated to describe inter-rater reliability of the selection process.

### Data extraction and synthesis

The following characteristics of the included studies were extracted through pretested data extraction forms: title, authors, journal, publication year, research field based on Journal Citation Reports (JCR) categories, country and city of study group, publication dates of included studies, publication dates of references, study population, intervention/exposure, outcome. The form was also used to extract the article’s main stress concept, as well as usage, verbatim descriptions and definitions or operationalizations of all included concepts.

Descriptive statistics and Cohen’s *κ* were analysed using IBM^®^ SPSS^®^ Statistics (version 28.0.1.0). JCR journal categories were grouped in general subjects. The verbatim description, operationalizations and definitions were merged and were systematically analysed to identify and cluster characteristics of concepts and to clarify their coherence. The clustering process was performed computer assisted using Microsoft Word^®^ and Microsoft Excel^®^ (Microsoft Office 365 version 2211). In case of ambiguity, the authors discussed until consensus was reached. Similar experiences used to describe a concept were grouped (e.g. physical or emotional bullying, gossiping, peer and sibling violence victimization and school violence were summarized as bullying). The overlap of the events was described by event-based concepts, and the overlap of characteristics of all concepts was visualized in Venn diagrams.

## Results

### Inclusion

**Figure 1.**
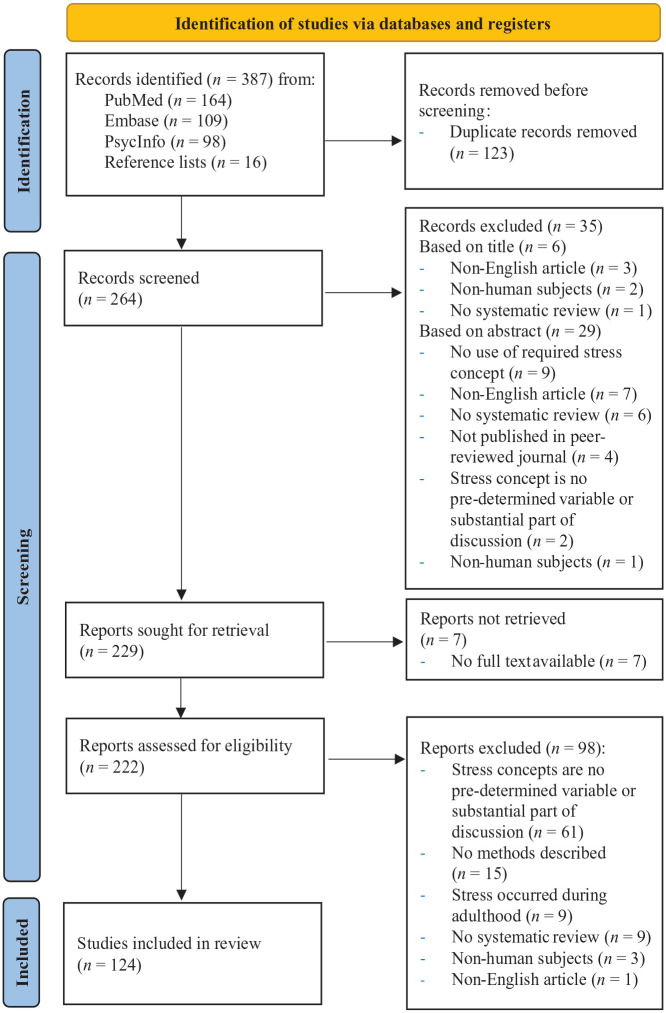
Flow diagram of inclusion process of the systematic review.

Our search yielded 387 records. After removing 123 duplicates, 264 titles and abstracts were screened for eligibility criteria, resulting in the exclusion of 35 articles. The remaining 229 articles were sought for retrieval, but seven full texts were unavailable. Of 222 remaining articles, 124 met the selection criteria. The selection process is shown in Figure 1. The inter-rater reliability of the selection process was substantial (*κ* = 0.749, *p* < 0.001). A *κ* < 0.6 is regarded as ‘substantial’ and *κ* < 0.8 as ‘almost perfect’. Initial disagreements between both reviewers were, for example, based on whether the concept was clearly a predetermined variable or was substantially used in the discussion, or whether the methods of an identified study were described completely and systematically.

### Study descriptives

The included studies were published between 2007 and 2021; the primary studies of the included reviews were published between 1963 and 2021, and their reference lists included studies published between 1921 and 2021. The study populations were diverse, for example, children, adults or people with specific diseases. Most research originated from North America (39.5%) and Europe (39.5%), followed by Australia (8.9%), South America (6.5%) and Asia (5.6%). The research groups originated from 22 different countries. Most studies were performed in the US (32.3%), England (14.5%) or Australia (8.9%). However, studies did not seem to be performed by the same research groups, as most studies were performed in different cities.

ACEs, CA and CT were the most-used concepts (69.4%, 73.4% and 68.5%, respectively). ACEs and CA were the main concept of a study most frequently, but ACEs were defined or operationalized most often (42.7%, compared with 26.6% and 23.4% for CA and CT, respectively). AL (14.5%) and TS (10.5%) were used in the least number of studies. Although CS was used more often (34%), AL, CS and TS were all rarely the main concept of a study (2.4%, 0.8% and 0.0%, respectively).

The journals in which studies were published were often described by multiple JCR categories. Most studies were published in psychological journals (58.9%), especially when their main concept was CA (66.7%), ACEs (45.8%), ELS (81.8%) or CT (85.7%). ACEs were the most frequently used main concept (47.6%). Of the studies where ACEs were the main concept, most were published in psychological fields (45.8%), but studies with ACEs as the main concept were also frequently published in other fields, like human sciences (27.1%), general medical fields (20.3%) and public and environmental health (18.6%). Only 11.3% of studies were published in public and environmental health journals. In public and environmental health journals, ACEs were most often the main concept.

In our dataset, the use of ACEs increased most in the last decade, but use of CA, CT and ELS increased as well. Table I shows how and how often the stress concepts were used; Table II shows the research fields they were used in; and Table III, their use through time.

**Table I.** Use of stress concepts in included studies, *n* (%): how often the concept was used in general and how often as main concept, and how often it was defined, operationalized or otherwise described.

The concept was:	CA	ACEs	AL	CS	ELS	CT	TS
Used in the study	91 (73.4%)	86 (69.4%)	18 (14.5%)	34 (27.4%)	34 (27.4%)	85 (68.5%)	13 (10.5%)
The main concept in the study	36 (29%)	59 (47.6%)	3 (2.4%)	1 (0.8%)	11 (8.9%)	14 (11.3%)	0 (0%)
Defined or operationalized in the study	33 (26.6%)	53 (42.7%)	5 (4.0%)	3 (2.4%)	7 (5.6%)	29 (23.4%)	1 (0.8%)
Otherwise described in study	58 (46.8%)	63 (50.8%)	12 (9.7%)	12 (9.7%)	15 (12.1%)	39 (31.5%)	6 (4.8%)

CA: childhood adversity; ACEs: adverse childhood experiences; AL: allostatic load; CS: chronic stress; ELS: early life stress; CT: childhood trauma; TS: toxic stress.

**Table II.** Research field where studies were published per main concept of the study, *n* (%): how (main) concepts were distributed over research fields based on JCR categories. One study could match multiple JCR categories.

Research field	Total	CA	ACEs	AL	CS	ELS	CT	TS
Psychological fields	73 (58.9%)	24 (66.7%)	27 (45.8%)	1 (33.3%)	0 (0%)	9 (81.8%)	12 (85.7%)	0 (0%)
Somatic medical fields^*^	26 (21.0%)	10 (27.8%)	9 (15.3%)	1 (33.3%)	0 (0%)	4 (36.4%)	2 (14.3%)	0 (0%)
Human sciences	26 (21.0%)	5 (13.9%)	16 (27.1%)	0 (0%)	0 (0%)	1 (9.1%)	4 (28.6%)	0 (0%)
General medical fields	16 (12.9%)	2 (5.6%)	12 (20.3%)	1 (33.3%)	1 (100%)	0 (0%)	0 (0%)	0 (0%)
Public and environmental health	14 (11.3%)	2 (5.6%)	11 (18.6%)	0 (0%)	0 (0%)	1 (9.1%)	0 (0%)	0 (0%)
Other	5 (4.0%)	1 (2.8%)	2 (3.4%)	0 (0%)	1 (100%)	0 (0%)	1 (7.1%)	0 (0%)

*Studies were mainly in neurosciences.

CA: childhood adversity; ACEs: adverse childhood experiences; AL: allostatic load; CS: chronic stress; ELS: early life stress; CT: childhood trauma; TS: toxic stress; JCR: Journal Citation Reports.

**Table III.** Number of times the concepts were used as main concept between 2007 and 2021.

Publication year	Total	CA	ACEs	AL	CS	ELS	CT	TS
2007–2009	2	1	0	0	0	0	1	0
2010–2012	7	4	0	1	0	2	0	0
2013–2015	13	3	4	1	0	2	3	0
2016–2018	28	12	9	0	1	2	4	0
2019–2021	74	16	46	1	0	5	6	0

CA: childhood adversity; ACEs: adverse childhood experiences; AL: allostatic load; CS: chronic stress; ELS: early life stress; CT: childhood trauma; TS: toxic stress.

### Descriptions of stress concepts

In the following section, the definitions, operationalization and use of each stress concept will be given, followed by otherwise-used descriptions and information from the included studies.

#### Chronic stress

CS was described as stress that persists for an extended period. The minimal duration to classify stress as chronic varied between studies and was often not specifically described. One study measured the self-reported frequency of CS behaviours in the last 3 months through the Trier Inventory of Chronic Stress [25]. Another assessed acute and CS over the past 6 months with the semi-structured UCLA Life Stress Interview [26].

CS in childhood as described in the reviewed articles, included stress accompanying ACEs [8,10,27,28], but could also originate from one’s (social) environment, like chronic exposure to poverty [8,29], substance abuse, violence [29], discrimination [8] or long-term caregiving for children or adults [29].

Short-termed stressors are associated with hyperactivation of the physiological stress response (e.g. elevated cortisol production) [26]. CS is described to lead to a sustained output of cortisol that can over time dysregulate the hypothalmic–pituitary–adrenal (HPA) axis [27,30]. Adaptations might initially serve temporary purposes. They can, however, have lifelong implications on the response to stressors [27]. Dysregulation of the HPA axis can diminish its activity and can weaken hormonal responses to stressors and lower diurnal cortisol levels [26,31], but it can also lead to exaggerated stress responses and a lower threshold for perceived stress [27]. A dysregulated HPA axis is found in many mental illnesses and might trigger or increase susceptibility to (stress-related) psychopathology during childhood and adulthood [27,32].

#### Toxic stress

TS was described as stress that results from frequent or chronic activation of the stress response, especially in the absence of support systems or protective factors, like stress resulting from exposure to childhood adversity [2,33-35]. TS can alter the function of physiological systems such as the (neuro)endocrine and immune system [33,36].

TS can lead to excess cortisol circulation [33]. An increased stress response can affect neural development, structure and function [34,36,37]. TS can particularly lead to remodelling of neurological pathways in the hippocampus, amygdala and prefrontal cortex [33]. Also, it can decrease a child’s stress threshold, increasing the child’s susceptibility to adverse reactions to traumatic experiences [35]. Through its impact on neurological, social, emotional and cognitive development, TS can lead to health conditions across the lifespan [34].

#### Allostatic load

AL was not used frequently, but Whelan et al. [8] described the operationalization of AL thoroughly. Stress is the foundation of the conceptual framework of AL. Whelan et al. described a consensus that, therefore, measurements of AL should include neuroendocrine and immunological biomarkers, as these systems participate in stress adaptation and affect health. The allostatic load index is an attempt to operationalize AL. The allostatic load index uses ten physiological measurements (physical measurements and markers in blood and urine) that can be transformed into summary scores that give an indication of physiological strain or successful adaptation in response to stressors. However, additions and revisions of the operationalization of AL have led to differences in used measures [8].

Allostasis is the adjustment of (neural, cardiovascular, autonomic, immune and metabolic) regulatory systems to acute physical and behavioural stressors. Short-term dysregulation of these systems results in physical and behavioural changes, bringing about adaptation to the stressor and promoting survival by maintaining internal stability [7,9,11,31,38,39]. The involved systems are highly integrated, so adjusting one system often results in responses in other systems [11]. Repeated or chronic stress can cause repeated adjustments of the involved systems [7,9,11,31]. AL describes this accumulated allostasis, that can potentially compromise the ability of these systems to function properly under normal circumstances. This can subsequently aggravate pathophysiology and lead to poor health outcomes throughout life due to pathologic stress responses [9-11,31,38].

#### Early life stress

Although there is not one agreed upon definition, ELS was often operationalized by a variety of adverse experiences [29,40-47] like poor parental caregiving or child maltreatment. Studies often described ELS to encompass traumatic experiences that occur during childhood and adolescence [29,42,46] but other operationalizations were being used as well. For example, LeMoult et al. [45] operationalized ELS more broadly as any event with characteristics that would be considered stressful during early life. ELS was typically used for stress in people younger than 18 years [44,48], sometimes described as ‘during childhood and adolescence’ [42,46], but could also be used to describe effects of perinatal, intrapartum, prenatal and maternal stress [32,49]. This variability emphasizes the importance of describing age during exposure, duration of exposure and severity of the stressor [40].

ELS was described to have detrimental effects on the developing brain, with effects continuing into adulthood [50], as it can affect behavioural, emotional, social, cognitive and physical development [46]. Early life represents a period of susceptibility to epigenetic alterations [32]. ELS is hypothesized to persistently modulate the activity of the HPA axis and the related stress response with a tendency towards hyperactivity [32]. Consequently, ELS may be involved in a predisposition to develop hyperarousal and stress hyperreactivity in adulthood, and ELS has been associated with vulnerability to numerous health problems (e.g. sleep problems, anxiety and mood regulation disorders) [32,43,51]. As ELS can elicit varying levels of cortisol dysregulation [40], cortisol has been used as a biological measure for ELS [10].

#### Childhood trauma

The Childhood Trauma Questionnaire (CTQ) was commonly used to assess childhood trauma [26,46,50,52-57]. The CTQ consists of a 25-item self-report scale on emotional, physical and sexual abuse, and emotional and physical neglect. CT was mostly self-reported retrospectively, making measurement prone to memory errors or recall bias [26,50,58-61]. The age of measurement usually ranged from 0 to 18 years [50,52,62-65] or during childhood [25,29,58,63,64,66] and adolescence [64]. Some studies used different age ranges, like an age below 16 years [52,57].

CT was often used but rarely defined. Van der Kolk was mentioned to have defined trauma as an emotionally painful event, that overwhelms a person’s ability to cope [67]. Siverns et al. noted that enduring repeated trauma increases the likelihood to experience multiple forms of trauma [68]. Trauma experienced early in life may have lifelong consequences across the major domains of personal and social functioning, with many survivors encountering the criminal justice and mental health systems [69].

#### Childhood adversity

CA was described as a broad condition marked by misfortune, and as the experience of social conditions or stressors that (are perceived to) threaten physiological equilibrium [70]. No gold standard for CA in early life has been established [71]. CA usually described exposure to severe acute or chronic stressors, that pose a severe risk for a child’s development [72]. Most measures included (different) sets of adverse experiences, relational and socioeconomic factors [22,26,70,71]. Some studies included either invalidated questions, expert observations, or public records, while others reported multiple effects of separate childhood adversities, without (the possibility to calculate) summarizing, global measures of trauma [71]. Usually CA was measured before 18 years of age [22,53,54,56,57,70,72-75], sometimes described as during childhood and adolescence [57,70,76]. Some studies used other criteria like occurring before the age of 11 and lasting for 6 months [77], before 16 years of age [25,29], before 19 years of age [78,79] or they also included prenatal exposure (until 18 years) [80-82].

Adversities often co-occur within individuals and can result in significant neurobiological, psychological or social strain that requires substantial adaptation from the child [26,72].

#### Adverse childhood experiences

ACEs were typically defined as negative, stressful or traumatic events in family environments that lack safety, stability or nurturing relationships [10,20]. ACEs are a concept regarding neglect, abuse and household dysfunction [1,2,7,10,11,27,28,30,33,48,60,61,67,83-98] and usually, at least the ten ACEs described by Felitti et al. [12] were used (i.e. physical, emotional and sexual abuse, physical and emotional neglect, separation of parents, domestic violence and having a household member suffering from a mental illness, substance abuse or that has been incarcerated). However, there appears to be no consensus on ACEs’ exact operationalization [35,87,99-101], and studies regularly modify this original scale to best fit their sample [61,87]. ACEs were usually operationalized as at least one specified exposure [67,84,88,100], using differing measurement tools, measuring different ACEs over different times and recall periods, reported by different informants [102]. ACEs were mostly assessed through self-report or informant reporting (mostly parents) [30,102,103] but were also extracted from police [103] or child protective services records [30]. This inconsistent operationalization across studies might complicate comparability [96,99,102]. ACEs were measured once in the first 19 years [2] but mostly in the first 18 years of life [7,10,11,21,61,63,83-87,99,100,102-105] or during childhood [1,10,11,30,34,35,37,63,83,88,89,101,105-109] and adolescence [1,37,60,63,105,106,108].

ACEs are typically associated with inadequate or inappropriate quality of care [88], that threatens the child’s familial [84,89,107], bodily or social safety or security [84]. ACEs occur frequently [101], are often severe [5,48] and chronic [30,35,38,48,92,101,110] and are interrelated, meaning that exposure to one ACE increases the likelihood of exposure to other ACEs [30,34,35,61,90,102,109,111,112]. Because ACEs inherently occur in childhood, they were mentioned as having a high risk of occurring during sensitive developmental periods [86,106]. ACEs are a dose-dependent risk factor [47,61,69,91,101,112,113] for the physical or psychological development, and threaten health [38,91,101,102,110]. ACEs’ lifelong consequences may influence all aspects of life, including relationships, parenting strategies and career decisions [69].

ACE counts are used often but have limitations. They usually do not account for the frequency, intensity and chronicity of exposures [21,22,30,96], the developmental timing during exposure, or for potential protective factors that might influence biological stress responses and health outcomes [21,30,89,96,102]. Therefore, ACE counts alone cannot explain the same ACEs leading to different outcomes, which potentially limits their usefulness for clinical decision-making purposes [96]. However, these limitations were mentioned as also likely contributing to the computational simplicity and widespread use of the concept in research [86].

### Overview of concepts

All concepts can refer to prolonged, repeated, interpersonal trauma from 0 to 18 years, for (often co-occurring) stressors in the family environment, social environment or neighbourhood. They can fundamentally alter physiological systems, resulting in significant health problems. There was no strict consensus on the operationalization of any of the concepts. Although all concepts can be used to describe harmful stress in early life, especially during sensitive developmental periods, AL, CS and TS are no childhood-specific concepts.

#### Psychophysiological model of stress

Each part of the psychophysiological model of stress (i.e. stress, stressor, stress response and stress effect) is relevant to each concept, but the concepts highlight different aspects of the model. CS mostly focuses on *stress*, as it emphasizes the persistent state of homeostatic challenge, with less specific focus on stressor, stress response or stress effect. ACEs, CA, CT and ELS focus most on experiences that can act as *stressors*. All stressor-oriented concepts, include a combination of physical stressors (e.g. physical abuse, having a serious illness or witnessing domestic violence) and psychological stressors (e.g. emotional neglect or mental illness in the household). One type of experience might act as a reactive and anticipatory stressor. For example, physical abuse can be a reactive stressor during the actual abuse and might cause conditioned stress responses afterwards. AL and TS focus more on the pathophysiological response to stressors. Of these concepts, AL seems to focus more on the measurable *stress response* that can subsequently lead to pathology. Although the stress response is an important aspect of TS as well, TS seems to specifically emphasize the *stress responses* with the potency to lead to harmful *stress effects*.

The psychophysiological model of stress can help identify differences between the concepts when choosing a concept for research purposes. More than the other aspects, the perception of being in a state of stress might be influenced by individual factors. This might cause concepts focusing on stress to be more subjective. Potential stressors can sometimes be measured objectively by observing the stressor. However, stressors are mostly surveyed, which means personal and cultural factors can influence whether the stimulus will be recalled and classified as a stressor. While physical threats might be more universal, perception of psychological stressors and (self-)stigma might be more influenced by personal or cultural differences [114]. Lastly, physiological stress responses and stress effects can be physically measured and therefore might be the most objective measure. However, stress responses and stress effects might also be affected by factors other than stress, like (genetic) predisposition, disease or lifestyle. Also, the possibility to measure stress responses retrospectively might be limited. And while some stress effects can also be surveyed, that would make them prone to the same limitations as mentioned for stressors.

#### Relationship between concepts

**Figure 2.**
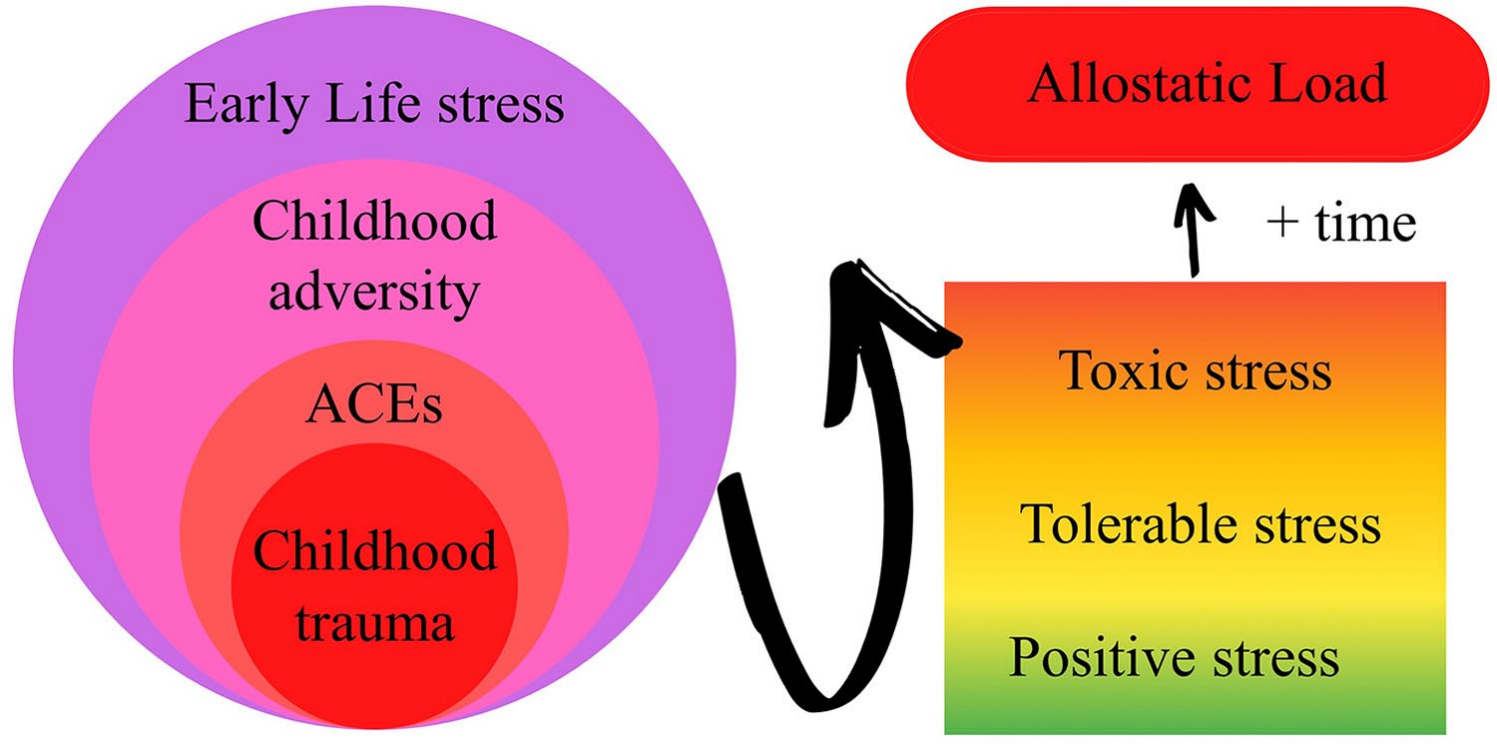
Model of relationship between different stress concepts. Chronic stress has not been included in this model. The possible protective effects of for example social support or resilience are not visualized. ACEs: adverse childhood experiences.

Multiple studies [33,34,37,95] mentioned that ACEs can lead to TS, especially when sustained over longer periods of time [35]. Although operationalized differently, all stressor-oriented concepts may negatively impact the same biological systems. Experiencing multiple, chronic, traumatic experiences, such as abuse and neglect during childhood might increase the risk of the stress response becoming toxic, which can contribute to AL through accumulating environmental insults on biological systems [31,39,49,77,115]. Figure 2 visualizes the relationship between the concepts.

#### Stressor-oriented concepts

**Figure 3.**
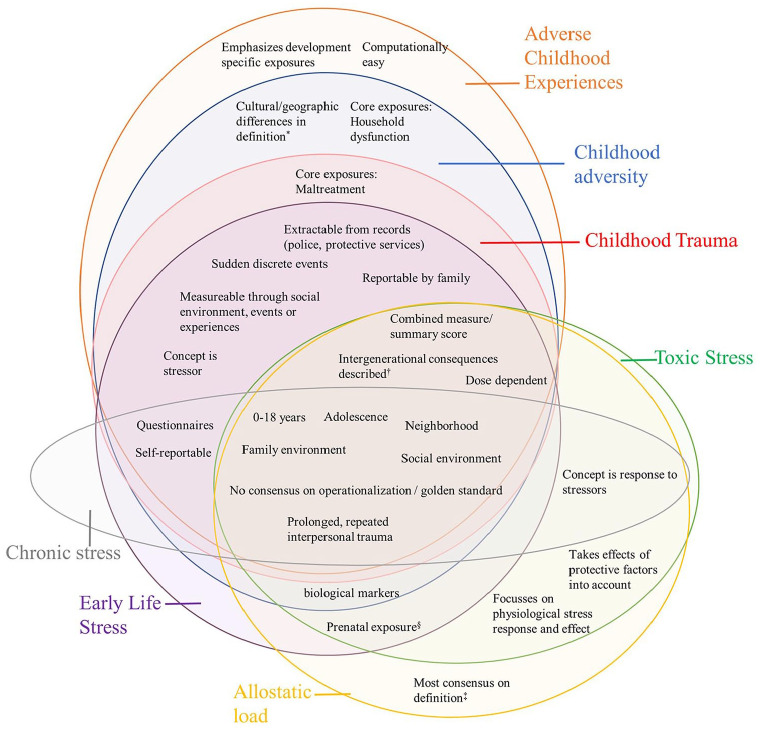
Venn diagram of the characteristics of seven stress concepts. ^*^Cultural and geographic differences are not mentioned for Early Life Stress given the broad definition of Early Life Stress. ^†^Intergenerational consequences are not mentioned explicitly for Allostatic Load but can be assumed through epigenetic changes. ^‡^Toxic Stress was seldomly defined, therefore statements about consensus on its definition are hard to substantiate. ^§^For Childhood Adversity prenatal exposure was mentioned incidentally but was usually absent.

Of the four predominantly stressor-oriented concepts, ELS had the broadest scope (e.g. it more often included prenatal stressors) and it was the only concept without an evident core set of stressors. Many studies on CT predate the ACE framework [86], and CT’s core stressors were often limited to child maltreatment. CA added household dysfunction to the core stressors. Although CA and ACEs were most similar, ACEs could be argued as a specific operationalization of adversity. Compared with CA, the concept of ACEs seemed to place a stronger emphasis to exposures in developmental environments that are usually sources of social support for children, like the school, family and home environment. The characteristics of the concepts are visualized in Figure 3.

**Figure 4.**
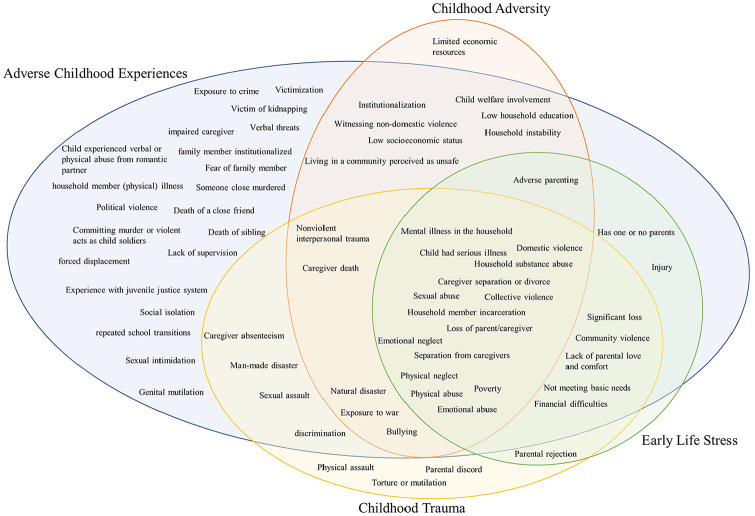
Venn diagram of experiences used to describe the stressor-oriented concepts.

Although not always as a core set of experiences, Felitti et al.’s [12] ten ACEs were used to operationalize all four stressor-oriented concepts. All concepts also described experiences other than Felitti’s ten ACEs. Other frequently used experiences like serious illness of the child, collective violence, loss of parent/caregiver and poverty were also used for all four concepts. ACEs were operationalized by the most experiences that were not used for other concepts. Many of these experiences were related to the household setting (death of sibling, fear of family member, forced displacement) or developmental situation (lack of supervision, repeated school transition), although other types of events were also described (e.g. victim of kidnapping, genital mutilation). Physical assault, parental discord and torture or mutilation were unique for CT. The unique experience for CA was limited economic resources. No experiences were used uniquely for ELS. Figure 4 visualizes the overlap of used experiences.

#### Stress concepts and study aims

The pathophysiological focus on stress responses might make TS and AL time consuming and costly to measure in larger populations. The stressor-oriented concepts and CS are often operationalized through surveys. Of these, CS was used the least and was operationalized less specifically and consistently. Of the stressor-oriented concepts, ELS seems to be the most broad and variable concept. Compared with CT, the inclusion of household dysfunction in the set of core experiences of CA and ACEs adds a dimension that seems relevant for public health. In recent years, according to our findings (Tables I–III), the use of ACEs has increased more than the use of other concepts, and it is currently being used most in general and in public health journals, specifically. So, although CA and ACEs might both be a suitable concept for public health, ACEs might be a preferred operationalization to use to increase uniformity and comparability with other studies.

## Discussion

Our study finds an overlap between the different stress concepts but also shows clear differences between them regarding their use, operationalization, specificity or emphasis in the psychophysiological stress model. ACEs’ measurement through social experiences makes operationalization feasible for large populations, and ACEs are well usable due to the relevant set of core experiences. The widespread use of ACEs in general, and in public and environmental health literature specifically, seems to have increased in recent years. Therefore, ACEs might be preferred in public health research.

Tables I–III show that the use of ACEs has grown significantly in childhood stress literature, and ACEs seem to have become the dominant concept used in this field in the past decade. Nevertheless, ACEs have not replaced other concepts, and the use of CA, AL and CT also continuously increased in this period. This emphasizes the value of being able to distinguish these concepts and to use the most fitting concept correctly.

The stressor-oriented concepts partially describe overlapping experiences but have distinctive characteristics. ELS seems more biology oriented than other stressor-oriented concepts and does not generally use a core set of events, as opposed to CA, ACEs and CT that become increasingly specific, respectively. Noteworthy is that ACEs, and probably all concepts, are evolving. For example, although Felitti et al.’s [12] ten-item ACE score is generally the fundament of the concept, many studies add or change experiences being measured. While many experiences not included in a specific ACE score could be defined as an ACE, the count of an altered set of ACEs cannot simply be used interchangeably with the original ACE score. By changing or adding ACEs to a score and therefore operationalizing the score differently, the interpretation of that ACE score will be altered. A broader operationalization might make the measured concept increasingly similar to the more general concept of CA. ACEs co-occur and might be seen as an indicator for total adversity, and it might not be feasible or necessary to use an exhaustive list of ACEs in any one score. Therefore, when using an ACE score, we suggest that authors clearly state which (part of a) score was used, for example, Felitti et al.’s ACE score [12] or the World Health Oraganization’s ACE-IQ [116]. When not adhering to a specified score, or when measuring other sets of ACEs, it might be more appropriate to refer to CA in general, to avoid misinterpretation.

In our review, the relatively high number of studies on ACEs should be considered when interpreting our results. This might lead, for example, to ACEs describing most different experiences. Also, strengths and limitations of ACEs were described often, and may therefore be described more explicitly in our results. However, it is likely that ACEs’ limitations also apply to other experience-based concepts. For example, other concepts do not usually account for frequency, intensity and chronicity of exposures either.

Frequently used experiences (e.g. Felitti et al.’s [12] ten ACEs) might seem to stabilize the concepts. However they are phrased inconsistently in different studies, which can lead to substantially different answers. For example, an affirmative answer to the questions whether someone has a family member, household member, caregiver or parent with a mental illness can all mean something different. This could influence variability significantly, especially combined with cultural and geographic influences on the perspective of ACEs (e.g. what constitutes neglect or mental illness), types of exposures (e.g. violence from civil war) and how health and social services access may mitigate the effects of ACEs, as suggested by Scott and colleagues [112]. A similar variability was seen in used age ranges: mostly the range of 0–18 years was used, but when the upper limit is the end of adolescence, this could be as high as 25 years. Due to the sensitive period of brain development during early development and adolescence, the relevance of including the prenatal and whole period of adolescence could be argued. Although Saarinen et al. [72] state the necessity to consider specific childhood adversities negatively affecting childhood development independent of age at exposure, others have suggested adopting methods that capture more information, particularly related to adversity type, timing and severity [22,115]. Objectivity and standardization in questioning is therefore warranted, and validated questionnaires should be used when available.

Of the two concepts focusing on the stress response and effect, AL was aimed more at measuring the stress response. AL was operationalized more thoroughly through physiological measures. Some things remain to be elucidated. It is, for example, possible that CS is not the only factor involved in AL [8]. Other factors possibly contributing to reactivity of the stress system and allostatic load are genes, early experiences and learned behaviours that reflect lifestyle choices such as diet, exercise and other habits [9]. TS on the other hand, was used more conceptually, and focused more on the ability of stress responses to cause negative stress effects. This concept was operationalized less through biomarkers, but neuroanatomical changes were described more specifically. The neurobiological focus might make these concepts more fitting for clinical settings.

We focused on stress-related concepts, but in daily life, stress and protective factors often coincide [117]. Because we found indications that severe stress can become toxic, especially in the absence of supportive relationships, we believe it meaningful to address protective factors like positive childhood experiences (PCEs) as well. PCEs represent positive experiences, for example, regarding parent–child attachment, family health and positive relationships [117,118]. Previous studies showed that positive, stimulating, home environments strengthen resilience [119], while ACEs were inversely correlated with resilience in adulthood [120]. If stressors are particularly relevant when social support is lacking, PCEs might aid in reducing toxic stress responses and prevent harm throughout life. If ACEs’ association with higher AL [121] is mediated through a TS response, being able to counteract TS responses could be highly relevant. Therefore, including PCEs in future research is advised for a more comprehensive and complete perspective on stress and its impact.

### Strengths and limitations

To our knowledge this is the first systematic review of literature to elucidate the use of different concepts used to study stress during childhood development, and to compare their use in literature.

Because of the large number of primary studies containing (one of) the search terms, including every primary study published on the selected concepts was not feasible. We therefore used systematic reviews instead of primary research articles, which led to a relevant number of included studies. Systematic reviews were assumed to use concepts based on their primary studies, thus representing those studies. Although we did not check whether the authors assessed the correct use of the concept in primary studies, we believe our methods valid for the following reasons. First, we only included studies from peer-reviewed journals as a primary quality control. Second, we only included studies where concepts were pre-determined or had a substantial place in the discussion of the article. The structured methods of a systematic review will often include a literature search to elucidate the included variables. Additionally, systematic reviews are often written by research groups that have expertise on the subject, and the acts of selecting and analysing studies and writing the review are likely to contribute to a well-founded view on included concepts. So, although our measurements depend on the interpretation of the authors of our selected reviews, we argue the concepts are well substantiated. Also, the primary studies covered by the reviews included in our review might overlap, while other studies might not be represented. If this led to a selection of studies, it is more likely that influential and high-quality studies are over-represented than the other way around. As high-quality studies might be expected to influence the way concepts are used more strongly, this is not expected to harm the results of our study.

For most concepts, especially stressor-oriented concepts, there are numerous measurement tools. As our aim was to elucidate concepts, rather than specific measurement tools, we focused on how authors interpreted and used the concepts based on the measurement tools in their research. Although it is possible that specific operationalizations and viewpoints on the concepts were omitted in this review, we expect that the most influential measures were represented.

Non-English studies were excluded, to eliminate the risk of missing terminology, missing nuances and mistranslating, as the methodology of our study would have required us to identify and translate the terminology from different languages to include them. This may have introduced selection bias. However, non-English studies were initially searched for and subsequently excluded. As this led to few exclusions, the impact of this criterion on our results is expected to be marginal.

Most studies were published in North America and Europe. Although we do not expect our results and conclusions to be substantially different in less-represented populations, some caution regarding generalizability might be warranted.

Database selection is another potential source of selection bias. Studies that were not included in used databases, might have been omitted. We decreased this risk by searching multiple databases: two large databases (PubMed and Embase) and one subject-focused database (PsychInfo). Additionally, we minimized the residual risk by checking all reference lists for additional sources.

Lastly, concepts that were used more than others could be substantiated more extensively. However, the less commonly used concepts were described and defined relatively well. Authors might have expected these concepts to be less known, leading them to provide more information.

## Conclusion

Different concepts used to describe stress during childhood development have distinctive characteristics, strengths and weaknesses. Their operationalizations, type of concept (stress, stressor, stress response or stress effect) and the fields in which they are published can support the choice for a concept. The most appropriate concept to use is likely dependent on the study or clinical context, and the choice of concept should be well considered. In public health research, stressor-oriented concepts may be most effective, as they can be well operationalized in large populations. Of these, ACEs might be the most fitting concept currently in public health.
